# N-Methyl D-aspartate receptor subtype 2B/Ca^2+^/calmodulin-dependent protein kinase II signaling in the lateral habenula regulates orofacial allodynia and anxiety-like behaviors in a mouse model of trigeminal neuralgia

**DOI:** 10.3389/fncel.2022.981190

**Published:** 2022-09-14

**Authors:** Zi-Fan Zhuang, Hong-Yun Wu, Ya-Yi Song, Lei Li, Xia Cui, Jie Yang, Xiang-Qing Xu, Wen-Qiang Cui

**Affiliations:** ^1^College of First Clinical Medicine, Beijing University of Chinese Medicine, Beijing, China; ^2^Department of Neurology, Affiliated Hospital of Shandong University of Traditional Chinese Medicine, Jinan, China; ^3^Department of Traditional Chinese Medicine, Lianyungang Maternal and Child Health Hospital, Lianyungang, China; ^4^Department of Cardiology, Affiliated Hospital of Shandong University of Traditional Chinese Medicine, Jinan, China

**Keywords:** trigeminal neuralgia, anxiety, lateral habenula, Ca^2+^/calmodulin-dependent protein kinase II (CaMKII), N-methyl-D-aspartate receptor (NMDAr)

## Abstract

Trigeminal neuralgia (TN) is a peripheral nerve disorder often accompanied by abnormalities in mood. The lateral habenula (LHb) plays important roles in the modulation of pain and emotion. In the present study, we investigated the involvement of the LHb in the mechanisms underlying allodynia and anxiety induced by partial transection of the infraorbital nerve (pT-ION) in mice. Our results indicated that pT-ION induced persistent orofacial allodynia and anxiety-like behaviors, which were correlated with increased phosphorylation of N-Methyl D-aspartate receptor (NMDAR) subtype 2B (p-NR2B) and Ca^2+^/calmodulin-dependent protein kinase II (p-CaMKII) in LHb neurons. Bilateral inhibition of NMDARs and CaMKII in the LHb attenuated the allodynia and anxiety-like behavior induced by pT-ION. Furthermore, bilateral activation of NMDARs in the LHb increased the expression of p-NR2B and p-CaMKII and induced orofacial allodynia and anxiety-like behaviors in naive mice. Adeno-associated virus (AAV)-mediated expression of hM3D(Gq) in CaMKII^+^ neurons of the bilateral LHb, followed by clozapine-N-oxide (CNO) administration, also triggered orofacial allodynia and anxiety-like behaviors in naïve mice with successful virus infection in LHb neurons (verified based on immunofluorescence). In conclusion, these findings suggest that activation of NMDA/CaMKII signaling in the LHb contributes to the occurrence and development of TN and related anxiety-like behaviors. Therefore, suppressing the activity of CaMKII^+^ neurons in the bilateral LHb by targeting NMDA/CaMKII may represent a novel strategy for treating pain and anxiety associated with TN.

## Introduction

Trigeminal neuralgia (TN), which has been considered among the most severe chronic pain disorders (Cruccu et al., 2020), is characterized by the experience of recurrent, transient, unilateral, and electric shock-like pain with abrupt termination (Cruccu et al., 2020). TN is one of the most common types of neuropathic pain, with an annual incidence of approximately 12.6 cases per 100,000 individuals (Jones et al., 2019). TN often affects the distribution of the second (maxillary) or third (mandibular) branch of the trigeminal nerve and can be triggered by daily sensory stimuli such as light touch, talking, chewing, tooth brushing, and cold air blowing across the face (Cheng et al., 2017). Excruciating orofacial pain often induces anxiety, which further exacerbates the sensation of pain (Bair et al., 2003). Unfortunately, because the mechanisms underlying the development of TN and TN-associated anxiety are unclear, effective treatments are currently unavailable.

The lateral habenula (LHb), a phylogenetically preserved brain structure within the superior colliculus, is one of the few brain regions that control both dopaminergic and 5-hydroxytryptaminergic (5-HT) neurotransmission (Wang and Aghajanian, 1977; Jhou et al., 2009). The dopaminergic and 5-HT systems play essential roles in the modulation of sensory and emotional responses. Thus, researchers have gradually recognized the LHb as a crucial regulator of pain and associated experiences of anxiety and depression (Li et al., 2016). Our previous study demonstrated that the LHb regulates pain and anxiety-like behaviors in a mouse model of TN (Cui et al., 2020b). Although recent studies also indicate that neuronal excitability in the LHb is strongly correlated with the occurrence and development of pain and emotional disorders (Browne et al., 2018; Dai et al., 2022), the underlying mechanisms remain unclear.

N-Methyl D-aspartate receptors (NMDARs) play an important role in the regulation of neuronal excitability. These glutamate receptors have long been considered to exhibit close associations with injurious stimulation and the maintenance of central sensitization (Furukawa et al., 2005; Kim et al., 2012; Xu et al., 2020). NMDARs play a key role in migraine, with higher glutamate concentrations and levels of NMDAR subtype 2B (NR2B) expression in patients with migraine (Tripathi et al., 2018). Research has also indicated that the NMDARs of LHb neurons are involved in the regulation of depression. Ketamine, an NMDAR antagonist, can rapidly relieve symptoms of depression by blocking the bursting activity of LHb neurons (Yang et al., 2018). NR2B is a key functional subunit of NMDARs, and its activation triggers NMDA channel opening, mediates inward calcium flow, and activates downstream Ca^2+^/calmodulin-dependent protein kinase II (CaMKII) (Bayer et al., 2001; Liu et al., 2020). This signaling cascade is an essential pathway involved in neuronal activation. Experimental research has identified CaMKII in the LHb as a key molecular determinant of depression (Li et al., 2013). Our previous study also demonstrated that inhibition of CaMKII^+^ neuronal excitability attenuates orofacial allodynia and related anxiety-like behaviors in a mouse model of TN (Cui et al., 2020b). However, whether NR2B/CaMKII signaling in the LHb is involved in the occurrence and development of TN and TN-related anxiety remains unclear.

In the present study, we aimed to examine the underlying role of NR2B/CaMKII signaling in CaMKII^+^ neurons of the LHb in the development of hyperalgesia following trigeminal nerve injury and related anxiety-like behaviors. To achieve this aim, we partially transected the infraorbital nerve (pT-ION) to establish a mouse model of TN, which was subjected to behavioral testing as well as chemicogenetic, immunofluorescence, and western blotting analyses. Our findings indicated that the NR2B/CaMKII pathway of the LHb contributed to the occurrence and development of orofacial pain and anxiety-like behaviors in pT-ION model mice.

## Materials and methods

### Animals

Adult male C57BL/6 mice (age: 7–9 weeks, weight: 22–25 g) were supplied by Beijing Vital River Laboratory Animal Technology Co., Ltd. (Beijing, China). Mice were acclimated to controlled conditions (22 ± 1°C, 12-h:12-h diurnal cycle, eight mice per cage, and water and food available *ad libitum*) for 1 week prior to experimental manipulation. The animal procedures of this study were performed in strict accordance with the standards of the National Institutes of Health Guide for the Care and Use of Laboratory Animals and the ethical standards of the International Association for the Study of Pain (Zimmermann, 1983). Extensive efforts were made to minimize the number of mice used and their suffering. Mice were randomly assigned to control or experimental groups for each experiment, and the sample size was determined based on our previous study (Cui et al., 2020b).

### Partially transected the infraorbital nerve model

The pT-ION surgery was performed as previously described (Cui et al., 2020a). Under intraperitoneal (i.p.) anesthesia with pentobarbital sodium (50 mg/kg), mice were placed on a surgical pad in the supine position, and the oral cavity was exposed. The ION was exposed by making a 5-mm-long incision in the left palatal-buccal mucosa and bluntly separating the tissue and blood vessels. The deep branches of the ION were tightly ligated with 4.0 catgut (BD171001, Shandong Bodac Co., Ltd., China) and severed from the distal end using surgical scissors, removing approximately 1 mm of nerve fibers to prevent regeneration. Great care was taken to avoid stretching of the ION. The wound was sealed with tissue glue. After surgery, the animals were anesthetized using an electric thermostatic blanket. In sham model mice, the ION was exposed without ligation or clipping. Postoperatively, potassium penicillin (50,000 units/kg/day, 14005, Jiangxi Keda Co., Ltd., China) was administered intramuscularly for three consecutive days. All operations were performed under sterile conditions, and no serious infections or postoperative complications were noted.

### Behavioral testing

Behavioral experiments were conducted by experimenters who were blinded to the grouping of the animals. All behavioral tests were conducted at a fixed time (9:00 A.M.–6:00 A.M.) in a quiet room held at a constant temperature (22 ± 1°C) and under soft lighting. The von Frey test and acetone test were performed to measure the mechanical and cold pain thresholds, respectively. The open field test (OFT) and elevated plus maze (EPM) tests were performed to assess anxiety-like behaviors in mice.

### Mechanical hyperalgesia test

The von Frey test was conducted as described in our previous study (Cui et al., 2020a). The ipsilateral hair was removed from the V2 (infraorbital area) and V3 (mandibular nerve area) areas using hair clippers (HC1066, Philips, The Netherlands). For acclimation, mice were individually placed in a box (8 cm × 8 cm × 10 cm) made of black metal mesh for 30 min on each of three consecutive days. Each von Frey filament (0.07, 0.16, 0.4, 0.6, 1.0, 1.4, and 2.0 g; Stoleting, United States) was applied to the skin of the V2 and V3 zones in ascending order. Each filament was applied five times with a constant predetermined force at intervals of a few seconds. To avoid tissue damage, a maximum of 6 s was set as the cutoff time. Rapid withdrawal of the head was considered a positive response. The size of the filament was defined as the pain threshold if it produced three positive responses over the five simulations.

### Cold hypersensitivity test

As previously described (Cui et al., 2020a), 50 μl of 90% acetone (diluted in distilled water) was drop-casted to the ipsilateral V3 skin using a 1-mL Hamilton microsyringe with a 25G needle to test for cold allodynia. To prevent acetone from entering the eyes of the mice, acetone was not injected into the V2 area. Mice were allowed to acclimate to the testing environment for 3 days, following which baseline values were measured. The total time spent wiping the orofacial area was recorded, and the cutoff time was set at 2 min. Cold allodynia was defined as an increase in the total wiping time when compared with baseline values or those observed in sham-operated animals. Special care was taken to avoid acetone contact with the skin or leakage during the test.

### Elevated plus maze test

The EPM test was performed as previously described to measure levels of anxiety-like behavior (Zheng et al., 2019). The maze contained a central platform (6 cm × 6 cm) and four arms (30 cm × 6 cm), including two open arms and two arms closed within walls with a height of 20 cm. The maze was elevated 40 cm from the floor. The light intensity was set at 15, 15, and 5 lx in the central area and in the open and closed arms, respectively. The temperature of the test environment was maintained at 22 ± 1°C. At the beginning of the experiment, one mouse was released at a time in the center area of the maze facing an open arm and allowed to explore freely for 5 min. Percentages of open-arm distance, open-arm entries, and open-arm time were calculated.

### Open field test

The OFT was performed as previously described (Zheng et al., 2019). Under controlled conditions [dim light (15 lx), 22 ± 1°C], mice were individually placed in the center of the open-air device, which was a box with a black floor measuring [50 cm × 50 cm × 40 cm (L × W × H)]. The central area was defined as a square with 25-cm sides. After 5 min of free exploration, the distance traveled in the central square, time spent in the central square, and number of crossings were recorded and analyzed.

### Virus injection and drug administration

To activate the CaMKII-expressing neurons of the LHb, mice were anesthetized and placed on a stereoscopic brain locator, following which 150 nL of the CaMKII-expressing neuron-targeting virus [pAAV overexpressing vector (pAOV)-CaMKII-HM3D(Gq)- EGFP-3FLAG] was injected at 10^12^ infectious units per ml (OBIO Technology Co., Ltd.) into the bilateral LHb (coordinates: bregma –1.62 mm, midline ± 0.45 mm, and skull surface –2.75 mm). Mice were allowed 3 weeks to recover and ensure stability of the transgene expression. The mice were then intraperitoneally injected with either saline or clozapine-N-oxide (CNO, 2.5 mg/kg, Sigma) approximately 45–55 min prior to behavioral testing. The hM3Dq receptor is a modified form of the human M3 muscarinic (hM3) activated by CNO and is involved in the Gq signaling pathway. Gq signaling facilitates the release of intracellularly stored calcium, which enhances neuronal excitability (Simonyi et al., 2005). Thus, the firing rate of hM3Dq-expressing neurons dramatically increases after CNO treatment. CaMKII promotes excitatory signaling involving glutamatergic neurons). Therefore, LHb injection of pAOV-CaMKII-HM3D(Gq)-EGFP-3FLAG virus followed by i.p. injection of CNO at specific time points results in enhanced excitability of glutamatergic neurons in the LHb. The virus was supplied by Obio Technology, Ltd. (Shanghai, China). To examine NMDAR and CaMKII signaling in pT-ION model mice, bilateral cannulation of the LHb was performed 21 days before the experiment. On the day of the experiment, the selective NMDAR agonist NMDA (50 ng, 150 nL, S7072, Selleck, United States) selective antagonist MK 801 (2 μg, 150 nL, S2876, Selleck, United States), and the specific, competitive CaMKII inhibitor KN93 (0.5 μg, 150 nL, S7423, Selleck, United States) were injected into the LHb. The administration dosage was determined based on the results of preliminary experiments. Behavioral tests were administered at particular points in time after drug injection.

### Western blotting

Under pentobarbital sodium anesthesia (50 mg/kg, i.p. injection), the habenula (Hb) of mice was quickly removed on ice and ultrasonically homogenized in RIPA lysis buffer (Beyotime, Shanghai, China). All samples were centrifuged at 12,000 rpm for 20 min at 4°C using a high-speed refrigerated centrifuge, following which the supernatant was separated. The protein concentration of the supernatant was measured using a Pierce bicinchoninic acid (BCA) kit (Thermo Fisher Scientific, Rockford, IL). Samples were mixed with protein loading buffer and boiled for 10 min. Total protein (30 μg) was separated via electrophoresis on a 10% polyacrylamide gel and transferred to a polyvinylidene fluoride (PVDF) membrane. The protein-PVDF membrane was blocked with 5% non-fat milk (dissolved in TBST solution [20 mM Tris-HCl, pH 7.5, 150 mM NaCl, and 0.05% Tween-20] for 1–2 h at normal temperature. The protein-PVDF membranes were incubated overnight with primary antibodies against p-NR2B (Tyr1472, 1:300 dilution, M2442, Millipore, United States), p-CaMKII (Thr286, 1:1,000 dilution, SC-12886-R, Santa Cruz Biotechnology, United States), NR2B (1:1,000 dilution, ab28373, Abcam, United Kingdom), CaMKII (1:1,000 dilution, 50049, Cell signaling technology, United States), c-FOS (1:1,000 dilution, ab208942, Abcam, United Kingdom) and GAPDH (1:10,000 dilution, 10494-1-AP, Proteintech, United Kingdom) at 4°C. The protein blots were washed three times with TBST solution and incubated with HRP-conjugated Affinipure Goat Anti-Rabbit IgG (H+L) secondary antibody (1:10,000 dilution, SA00001-2, Proteintech) for 1–2 h at normal temperature. The blot images were captured using an ImageQuant LAS4000 mini-image analyzer (GE Healthcare, United Kingdom). Quantity One software (version 4.6.2, United States) was used to analyze blot intensities.

### Immunofluorescence

Immunofluorescence (IF) analyses were performed to observe the localization of viral transfection in the LHb. Mice injected with virus were perfused with 0.9% NaCl solution followed by ice-cold 4% paraformaldehyde (prepared in 0.1 M PBS solution) under pentobarbital sodium anesthesia. Brain tissue was separated and post-fixed overnight in 4% paraformaldehyde at 4°C. The tissue samples were then soaked in 20% sucrose solution for 24 h and 30% sucrose solution for 3–5 days for dehydration, following which they were sectioned into 30 μm-thick slices using a freezing microtome (Leica 2000, Germany). The slices were washed with PBST (PBS solution with 0.3% Triton X-100) three times and then coverslipped with 4′,6-diamidino-2-anilinol (DAPI) Fluorosan-G^®^ (0100-20, Southern Biotechnology, United States). The IF images were captured using a multiphoton laser dot scanning confocal microscope system (FV1000; Olympus, Japan).

### Statistical analysis

GraphPad Prism software 6.0 (San Diego, CA, United States) was used to analyze the data. All data are expressed as the mean ± standard error of the mean (SEM). Student’s *t*-test or one-way analyses of variance (ANOVA) were used to analyze group differences, followed by Dunnett’s *post hoc* multiple comparison test or Tukey’s multiple comparison test. Two-way repeated-measures ANOVA followed by Tukey’s multiple comparison test were performed to analyze the time courses of mechanical hyperalgesia and cold allodynia. Statistical significance was set at *p* < 0.05.

## Results

### Partially transected the infraorbital nerve induces orofacial pain and anxiety-like behaviors in mice

**FIGURE 1:**
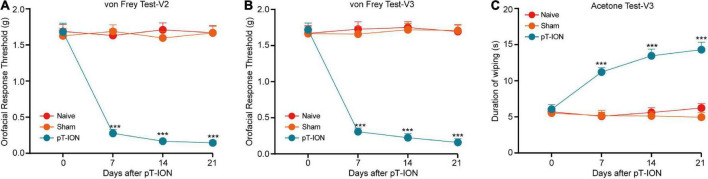
The time course of chronic orofacial pain after pT-ION surgery. Persistent primary hyperalgesia in the V2 area **(A)** and secondary hyperalgesia in the V3 area **(B)** induced by pT-ION was shown as the reduced threshold to von Frey stimulus. Long-lasting secondary cold allodynia in the V3 area **(C)** induced by pT-ION was shown as an increased duration of wiping in response to acetone stimulus. *N* = 8 mice per group, *** *p* < 0.001 vs. Naive.

The trigeminal nerve consists of three branches: the ophthalmic nerve (innervating the V1 region of the orofacial region), the maxillary nerve (V2 region), and the mandibular nerve (V3 region) (Kim et al., 2014). Consistent with our previous study (Cui et al., 2020a), pT-ION model mice exhibited primary mechanical nociceptive hypersensitivity in the V2 area innervated by the damaged ION and secondary mechanical hypersensitivity in the V3 region innervated by the uninjured mandibular nerve. On postoperative day 7, pT-ION mice exhibited a significant reduction in the V2 response threshold on the ipsilateral flank, which persisted for at least 21 days [two-way ANOVA and Tukey’s test, *F*_(2,__21)_ = 25.56, *p* < 0.0001; Figure 1A]. Similar reductions were observed in the V3 area with secondary injury over the same time frame [two-way ANOVA and Tukey’s test, *F*_(2,__21)_ = 88.81, *p* < 0.0001; Figure 1B]. In addition, model mice exhibited cold nociceptive hypersensitivity in the V3 region during the acetone test, which persisted for 21 days postoperatively [two-way ANOVA and Tukey’s test, *F*_(2,__21)_ = 17.99, *p* = 0.0003; Figure 1C], suggesting that pT-ION induced secondary cold nociceptive hypersensitivity in mice.

**FIGURE 2:**
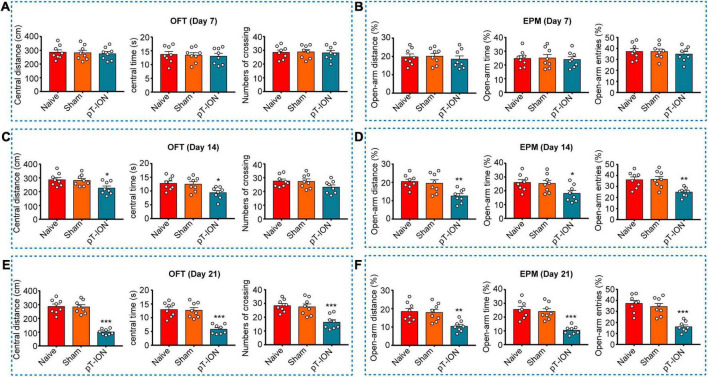
The OFT and EPM test of mice on post-pT-ION day 7, 14, 21. The central distance **(A)**, central time **(B)**, numbers of crossing **(C)** in the OFT test, and the percentage of open-arm distance **(D)**, open-arm time **(E)**, and open-arm entries **(F)** in the EPM test were measured on post-pT-ION day 7, 14 and 21. *N* = 8 mice per group, *p < 0.05, **p < 0.01, and ***p < 0.001 vs. Naive.

To explore whether pT-ION induces anxiety-like behavior in mice, we performed the OFT and EPM test on postoperative days 7, 14, and 21. Consistent with our previous study (Cui et al., 2020b), the OFT test showed that at day 14 and 21 after pT-ION, the mice traveled shorter distances (one-way ANOVA and Dunnett’s test; Day 14: *F* = 4.679, *p* = 0.0209; Day 21: *F* = 51.55, *p* < 0.0001), spent less time in the central region (one-way ANOVA and Dunnett’s test; Day 14: *F* = 4.943, *p* = 0.0174; *F* = 23.47, *p* < 0.0001; Figures 2C,E) than Naive mice, but not at day 7 (one-way ANOVA and Dunnett’s test; central distance: *F* = 0.1015, *p* = 0.9039; central time: *F* = 0.0975, *p* = 0.9075; Figure 2A). At day 21 after surgery, pT-ION mice made fewer central crossings (one-way ANOVA and Dunnett’s test, *F* = 13.01, *p* = 0.0002, Figure 2E) than Naive mice, but not at day 7 (one-way ANOVA and Dunnett’s test, *F* = 0.0475, *p* = 0.9537, Figure 2A) and 14 (*F* = 2.282, *p* = 0.1268, Figure 2C). In the EPM test, at day 14 and 21 after pT-ION, model mice exhibited decreases in percentage values for open arm distance (one-way ANOVA and Dunnett’s test; Day 14: *F* = 7.894, *p* = 0.0028; Day 21: *F* = 8.357, *p* = 0.0021), time (one-way ANOVA and Dunnett’s test; Day 14: *F* = 4.019, *p* = 0.0333; Day 21: *F* = 19.82, *p* = 0.0021), and entries (one-way ANOVA and Dunnett’s test; Day 14: *F* = 8.276, *p* = 0.0022; Day 21: *F* = 21.43, *p* < 0.0001; Figures 2D,F) when compared with Naive mice, but not at day 7 (one-way ANOVA and Dunnett’s test; distance: *F* = 0.2741, *p* = 0.7630; time: *F* = 0.0570, *p* = 0.9447; entries: *F* = 0.3034, *p* = 0.7415; Figure 2B). These results demonstrate that partial damage of the trigeminal nerve is sufficiently aversive to trigger anxiety-like behavior in mice.

### Phosphorylation of N-Methyl D-aspartate receptor subtype 2B and Ca^2+^/calmodulin-dependent protein kinase II in the habenula is upregulated in partially transected the infraorbital nerve model mice

**FIGURE 3:**
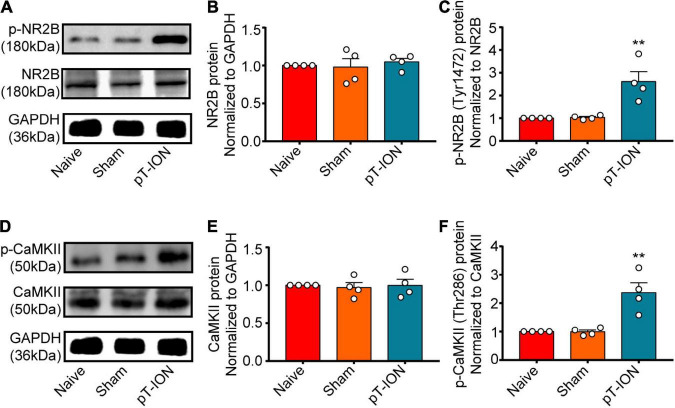
The expression of p-NR2B and p-CaMKII on post-pT-ION day 21. The NR2B, p-NR2B, CaMKII and p-CaMKII protein expression in the Hb was shown by the representative blots **(A,D)**. The quantitative results were shown by the histograms **(B,C,E,F)**. *N* = 4 mice per group, **p < 0.01 compared with the Naive group.

The phosphorylation of NR2B reflects the activation of NMDARs. CaMKII is a downstream target of NMDA, and its phosphorylation is a marker of neuronal activation. Western blotting was performed to evaluate levels of p-NR2B and p-CaMKII expression in the Hb at day 21 after pT-ION. Levels of both p-NR2B (one-way ANOVA and Dunnett’s test, *F* = 12.82, *p* = 0.0023, Figures 3A,C) and p-CaMKII (one-way ANOVA and Dunnett’s test, *F* = 15.20, *p* = 0.0013, Figures 3D,F) expression were significantly higher in the pT-ION group than in the Naive group, whereas the levels of NR2B (one-way ANOVA and Dunnett’s test, *F* = 0.2438, *p* = 0.7886, Figures 3A,B) and CaMKII (one-way ANOVA and Dunnett’s test, *F* = 0.0761, *p* = 0.9273, Figures 3D,E) had no significant changes in pT-ION group than in the Naive group. These results suggested that the NR2B/CaMKII signaling of Hb was activated after ION injury.

### Inhibition of N-Methyl D-aspartate receptors and Ca^2+^/calmodulin-dependent protein kinase II in the lateral habenula attenuates partially transected the infraorbital nerve –induced orofacial hyperalgesia and anxiety-like behavior

**FIGURE 4:**
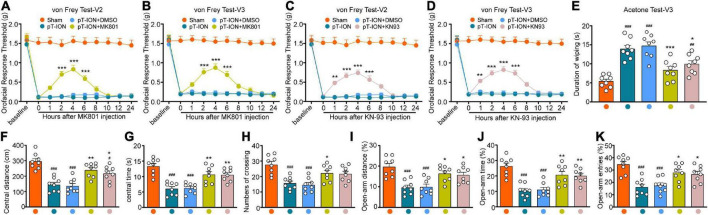
The orofacial allodynia and anxiety-like behavior after MK801 and KN-93 administration. The mechanical hypersensitivity in both V2 and V3 areas were attenuated by LHb administration of the NMDA receptor antagonist MK801 (from 2 to 6 h after injection; **A,B**) and CaMKII inhibitor KN-93 (from 1 to 6 h after injection; **C,D**) at day 21 after pT-ION. In acetone test, The increased duration of wiping induced by pT-ION was decreased by MK801 and KN-93 at 4 h after injection **(E)**. The reduced central distance **(F)**, central time **(G)**, numbers of crossing **(H)** in the OFT and the reduced percentage of open-arm distance **(I)**, open-arm time **(J)**, and open-arm entries **(K)** in EPM test induced by pT-ION were significantly reversed at 4 h after LHb injection of MK801 and CaMKII. *N* = 8 mice per group, ^#^*p* < 0.05, ^##^*p* < 0.01, ^###^*p* < 0.001 vs. the Sham group; *p < 0.05, **p < 0.01, ***p < 0.001 vs. the pT-ION+DMSO group.

MK801—a potent, non-competitive, specific antagonist of NMDARs—acts at the cell membrane (Okada et al., 2019). KN-93 is a cell-permeable and competitive reversible CaMKII inhibitor (Pellicena and Schulman, 2014). Behavioral tests were used to examine the effect MK801 and KN93 injection into the LHb at day 21 after nerve injury. The orofacial response thresholds of the V2 and V3 regions were significantly elevated within 6 h after MK801 or KN-93 injection, peaking at around 4 h, when compared with those in the pT-ION group [MK801-von Frey Test-V2: two-way ANOVA and Tukey’s test, *F*_(3,__28)_ = 15.63, *p* < 0.0001; MK801-von Frey Test-V3: two-way ANOVA and Tukey’s test, *F*_(3,__28)_ = 13.39, *p* < 0.0001; KN-93-von Frey Test-V2: two-way ANOVA and Tukey’s test, *F*_(3,__28)_ = 14.86, *p* < 0.0001; KN-93-von Frey Test-V3: two-way ANOVA and Tukey’s test, *F*_(3,__28)_ = 14.34, *p* < 0.0001; Figures 4A–D]. Cold nociceptive hypersensitivity was also significantly attenuated by both MK801 and KN93 at 4 h after injection [one-way ANOVA and Tukey’s test, *F* = 18.62, *p* < 0.0001; Figure 4E). These mice traveled longer distances (one-way ANOVA and Tukey’s test, *F* = 16.27, *p* < 0.0001, Figure 4F), spent more time in the central region (one-way ANOVA and Tukey’s test, *F* = 15.63, *p* < 0.0001, Figure 4G), and made more central crossings (one-way ANOVA and Tukey’s test, *F* = 9.29, *p* < 0.0001, Figure 4H) in the OFT at 4 h post-injection, when compared with pT-ION group. Meanwhile, the percentage values for open arm distance (one-way ANOVA and Tukey’s test, *F* = 9.94, *p* < 0.0001, Figure 4I), time (one-way ANOVA and Tukey’s test *F* = 13.70, *p* < 0.0001, Figure 4J), and the number of entries (one-way ANOVA and Tukey’s test, *F* = 10.62, *p* < 0.0001, Figure 4K) in the EPM test also increased following injection of either MK801 or KN93. No significant differences were observed between the pT-ION+DMSO and pT-ION groups (Figures 4A–K).

### Injection of N-Methyl D-aspartate receptor into the lateral habenula increases phosphorylation of N-Methyl D-aspartate receptor subtype and Ca^2+^/calmodulin-dependent protein kinase II and leads to orofacial hyperalgesia and anxiety-like behaviors

**FIGURE 5:**
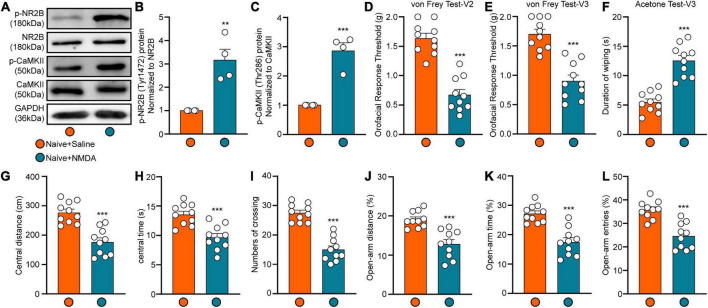
The expression of p-NR2B and p-CaMKII and behavior results after LHb injection of NMDA. The p-NR2B and p-CaMKII protein expression of the Hb at 4 h after NMDA injection was shown by the representative blots **(A)**. The quantitative results were shown by the histograms **(B,C)**, *N* = 4 mice per group. The mechanical and cold allodynia in both V2 and V3 areas appeared at 2 h after LHb NMDA administration **(D–F)**. The central distance **(G)**, central time **(H)**, numbers of crossing **(I)** in the OFT and the percentage of open-arm distance **(J)**, open-arm time **(K)**, and open-arm entries **(L)** in EPM test were significantly decreased at 2 h after LHb injection of NMDA. *N* = 10 mice per group, **p < 0.01 and ***p < 0.001 compared with the Naive+Saline group.

To investigate whether activation of NMDARs (particularly NR2B) in the LHb triggers trigeminal pain and anxiety-like behaviors, we injected NMDA into the LHb of naïve mice via a pre-buried casing. Western blotting and behavioral tests were performed 2 h after injection. Western blotting results showed that levels of both p-NR2B and p-CaMKII expression were significantly increased following NMDA injection (Student’s *t*-test, p-NR2B: *t* = 4.69, *p* = 0.0034; p-CaMKII: *t* = 6.76, *p* = 0.0005; Figures 5A–C), suggesting significant activation of NMDARs and CaMKII in the LHb of naïve mice. When compared with those observed in the Naive+Saline group, the orofacial response thresholds in both the V2 and V3 regions observed in the Naïve +NMDA group were significantly decreased in the von Frey test (Student’s *t*-test, von Frey Test-V2: *t* = 7.82, *p* < 0.0001; von Frey Test-V3: *t* = 6.25, *p* < 0.0001; Figures 5D,E), while the duration of wiping was significantly increased in the acetone test (Student’s *t*-test, Acetone Test-V3: *t* = 7.01, *p* < 0.0001; Figure 5F). In addition, Naive+NMDA mice exhibited decreases in the central distance traveled (Student’s *t*-test, *t* = 5.49, *p* < 0.0001; Figure 5G), time spent in the central region (Student’s *t*-test, *t* = 4.47, *p* = 0.0003; Figure 5H), and number of central crossings (Student’s *t*-test, *t* = 7.95, *p* < 0.0001; Figure 5I) in the OFT test when compared with Naive+Saline mice. The percentage values for open-arm distance (Student’s *t*-test, *t* = 4.87, *p* < 0.0001; Figure 5J), time (Student’s *t*-test, *t* = 5.56, *p* < 0.0001; Figure 5K), and the number of entries (Student’s *t*-test, *t* = 5.28, *p* < 0.0001; Figure 5L) in the EPM test were also lower for Naïve+NMDA mice than for Naïve+Saline mice. These results suggest that NMDAR activation in the LHb mediates CaMKII activation, which in turn triggers orofacial hyperalgesia and anxiety-like behavior.

### Activation of Ca^2+^/calmodulin-dependent protein kinase II^+^ neurons in the lateral habenula induces orofacial hyperalgesia and anxiety-like behaviors

**FIGURE 6:**
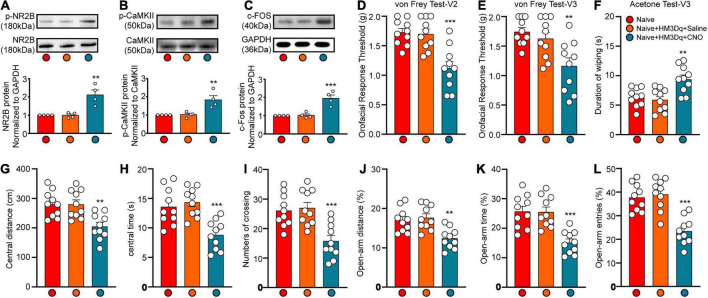
Chemicogenetic activation of bilateral LHb neurons triggered orofacial pain and anxiety-like behavior. The c-FOS, p-NR2B and p-CaMKII protein expression of the Hb at 45 min after CNO injection was shown by the representative blots **(A–C)**. The mechanical and cold allodynia were triggered by bilateral pAOV-CaMKII-HM3D(Gq)-EGFP-3FLAG injection into the LHb neurons and the subsequent CNO administration **(D–F)**. The central distance **(G)**, central time **(H)**, and numbers of crossing **(I)** in the OFT and the percentage of open-arm distance **(J)**, open-arm time **(K)**, and open-arm entries **(L)** in EPM test were significantly reduced by the activation of LHb neurons by HM3Dq-CNO. *N* = 10 mice per group, **p < 0.01, ***p < 0.001 vs. the Naive+HM3Dq+Saline group.

**FIGURE 7:**
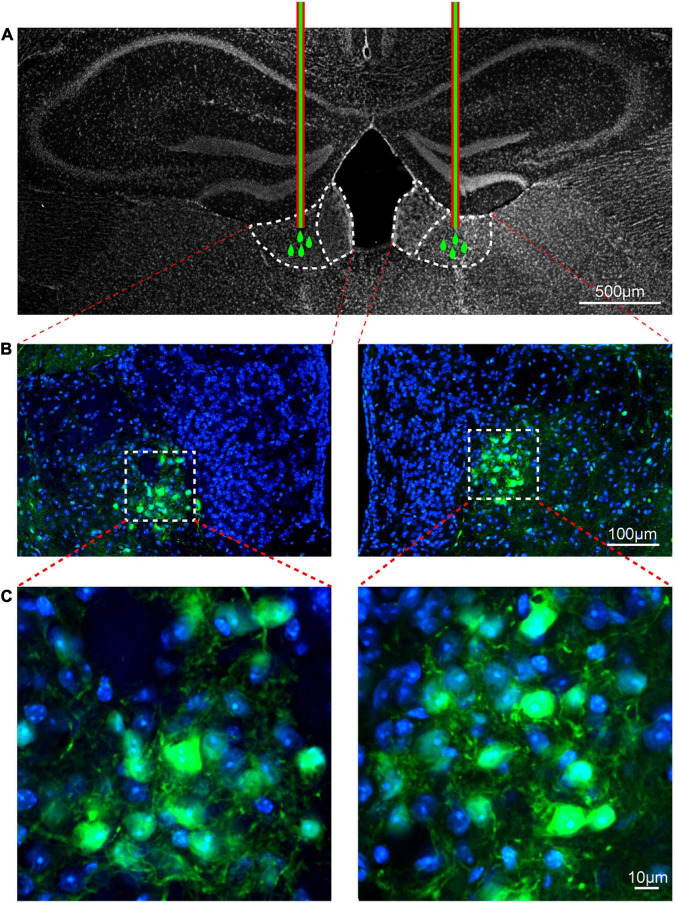
Viral transfection was mainly localized in the lateral habenula. The injection locations of pAOV-CaMKII-HM3D(Gq)-EGFP-3FLAG virus **(A)**. The virus transfection position in the LHb was shown by the typical immunofluorescence staining; Green, EGFP; Blue, DAPI [**B,C** (Zoom)]. Scale bar: 500 μm **(A)**, 100 μm **(B)** and 10 μm **(C)**.

To demonstrate the role of CaMKII^+^ neurons in the LHb in orofacial hyperalgesia and related anxiety-like behaviors, we injected a virus targeting CaMKII-expressing neurons [pAOV-CaMKII-HM3D(Gq)-EGFP-3FLAG] into the bilateral LHb of naive mice. At day 21 post-injection, CNO was injected (2.5 mg/kg, intravenously) to activate CaMKII^+^ neurons. Behavioral tests were performed 45 min after CNO injection, following which western blotting was perform to observe the expression of p-NR2B, p-CaMKII, and the neuronal excitability marker c-Fos. IF was performed to reveal the localization of viral infection. The western blot results showed that the expression of p-NR2B, p-CaMKII, and c-FOS increased significantly after CNO injection when compared with that after saline injection (p-NR2B: one-way ANOVA and Tukey’s test, *F* = 15.72, *p* = 0.0012; p-CaMKII: one-way ANOVA and Tukey’s test, *F* = 11.68, *p* = 0.0032; c-FOS: one-way ANOVA and Tukey’s test, *F* = 21.67, *p* = 0.0004; Figures 6A–C). The behavioral test results showed that the mechanical stimulation thresholds in V2 (one-way ANOVA and Dunnett’s test, *F* = 14.60, *p* < 0.0001, Figure 6D) and V3 (one-way ANOVA and Dunnett’s test, *F* = 7.97, *p* = 0.0019, Figure 6E) decreased, while the duration of wiping induced by acetone stimulation increased in V3 (one-way ANOVA and Dunnett’s test, *F* = 9.38, *p* = 0.0008, Figure 6F) after CNO injection. Meanwhile, when compared with mice in the Naïve+HM3Dq+Saline group, those in the Naive+HM3Dq+CNO group exhibited significant decreases in the central distance traveled (one-way ANOVA and Dunnett’s test, *F* = 9.46, *p* = 0.0008, Figure 6G), time spent in the central area (one-way ANOVA and Dunnett’s test, *F* = 12.49, *p* = 0.0001, Figure 6H), and the number of central crossings (one-way ANOVA and Dunnett’s test, *F* = 11.46, *p* = 0.0002, Figure 6I) in the in the OFT. These mice also exhibited decreases in percentage values for open-arm distance (one-way ANOVA and Dunnett’s test, *F* = 9.71, *p* = 0.0007, Figure 6J), time (one-way ANOVA and Dunnett’s test, *F* = 15.17, *p* < 0.0001, Figure 6K), and the number of entries (one-way ANOVA and Dunnett’s test, *F* = 21.22, *p* < 0.0001, Figure 6L) in the EPM test when compared with those in the Naive+HM3Dq+Saline group. The IF images showed that the virus was primarily located in LHb neurons (Figures 7A–C). These results indicate that activation of CaMKII^+^ neurons in the LHb via HM3Dq-CNO chemicogenetic methods is sufficient to evoke orofacial hyperalgesia and anxiety-like behaviors in naive mice.

## Discussion

Allodynia caused by TN is often severe and unpredictable, causing patients to develop a fear of unpredictable pain, anxiety, and even depression (Macianskyte et al., 2011). However, recent research on the mechanisms underlying TN has mainly focused on pain while ignoring the emotional response induced by TN. This approach limits our understanding of the mechanisms underlying TN and hinders the development of effective pharmacological treatment strategies. Our previous study demonstrated that the LHb is involved in the occurrence and development of pain and the related anxiety induced by TN (Cui et al., 2020b). Based on this research, we further investigated the mechanisms by which the LHb contributes to pain and anxiety in cases of trigeminal nerve injury.

Our results indicated that pT-ION induced long-lasting primary mechanical hypersensitivity in the V2 area, as well as secondary mechanical and cold allodynia in the V3 area (Figure 1). As the pain persisted, pT-ION mice began to exhibit anxiety-like behavior on day 14 after nerve injury. However, not all differences had reached significance at this time, with some indicators only exhibiting a trend toward significance (Figure 2). However, pT-ION mice exhibited significant and stable anxiety-like behaviors on day 21 after surgery (Figure 2). The time course of changes in p-NR2B and p-CaMKII expression in the LHb after pT-ION was in accordance with the behavioral chronology (Supplementary Figure 1). Thus, we chose the 21-day time point for subsequent experiments when investigating the role of the LHb in generating the anxiety-like behaviors induced by pT-ION.

The majority of NMDA receptors consist of the necessary structural subunit NR1 and the functional subunit NR2, which is divided into four types: NR2A, NR2B, NR2C, and NR2D. Several studies have demonstrated the involvement of all four types of the NR2 subunit in neuropathic pain (Yang et al., 2014; Jing et al., 2022; Zhang et al., 2022). The NR2B subunit is the focus of chronic pain research, as it plays an essential role in the functional activity of NMDARs at the cell surface and in the development of central sensitization, a classical feature of chronic pain (Chen et al., 2018; Zhang et al., 2020). Recent studies have indicated that the NR2B subunit is involved in mechanical and cold pain induced by inflammation and nerve damage via KIF17/mLin10/NR2B signaling (Uniyal et al., 2021, 2022a,2022b). Moreover, previous studies have documented the role of the NR2B subunit in neurons of the spinal cord and trigeminal spinal subnucleus caudalis in the context of TN (Gu et al., 2012; Kimura et al., 2022). However, the role of the NR2B subunit in the LHb remains poorly understood. Our findings indicate that pT-ION triggered increased activation of the NR2B subunit in NMDA receptors of the Hb (Figure 3). Meanwhile, the mechanical and cold allodynia induced by pT-ION were blocked by injection of the NMDA receptor antagonist MK801 into the LHb (Figure 4). These results indicate that the NR2B subunit of NMDARs in the LHb contributes to TN induced by pT-ION.

NMDARs are glutamate receptors that play an important role in the excitatory regulation of LHb neurons (Valentinova and Mameli, 2016). Several recent animal experiments and clinical studies (Amidfar et al., 2019; Abdallah et al., 2020) have consistently demonstrated that excitatory signaling mediated by NMDARs regulates mood and that NMDAR antagonists produce rapid and long-lasting antidepressant effects. One study reported that ketamine exerts a rapid antidepressant effect by blocking NMDAR-dependent LHb bursts (Yang et al., 2018). The NR2B subunit is highly expressed in the LHb, and NR2B activation-dependent long-term synaptic potentiation (LTP) of LHb neurons contributes to the occurrence and development of depression following exposure to stress (Park et al., 2017; Kang et al., 2020). Consistent with these results, we observed that both increased expression of p-NR2B induced by pT-ION and injection of NMDA into the LHb induced anxiety-like behavior in mice (Figures 2, 3, 5). Notably, injection of the NMDA antagonist MK801 rapidly relieved anxiety-like behaviors induced by pT-ION (Figure 4).

Some studies have indicated that targeting NMDA with blockers may produce side effects such as headache and motor impairment (Cohen et al., 2006; Short et al., 2018). However, these effects may be closely related to the method and dose of administration, with systemic administration more likely to trigger certain effects. To eliminate such interference, we examined the orofacial pain threshold and limb motor function in naive mice 4 h after injection of MK801 into the LHb. Our results indicated that injection of the NMDA antagonist MK801 into the LHb produced no effects on the orofacial pain threshold or motor ability in naïve mice (Supplementary Figure 2). Therefore, selective inhibition of LHb function using NR2B-targeting methods may represent a therapeutic approach to TN and TN-related negative emotions.

All four CaMKII isoforms (α, β, γ, and δ) are present in the brain tissue. Among these, CaMKIIα and CaMKIIβ are predominantly found in the neurons and are highly expressed at excitatory synaptic sites in the brain. Increasing CaMKIIβ expression in the LHb observably enhances the efficacy of synaptic transmission and spike output from LHb neurons, leading to profound depressive-like symptoms, while no such effects are observed upon increasing CaMKIIα expression (Li et al., 2013). Consistent with these findings, we observed no significant increases in CaMKIIα expression, although levels of CaMKIIα phosphorylation were significantly increased after pT-ION modeling in the preliminary experiment (Figure 3). One study demonstrated that M4-type melanopsin-expressing retinal ganglion cells innervate GABAergic neurons in the thalamic ventral lateral geniculate nucleus and intergeniculate leaflet, causing the inhibition of CaMKIIα neurons in the LHb that underlies the antidepressant effects of light therapy (Huang et al., 2019). In this study, the anxiety-like behavior induced by pT-ION was also attenuated by KN93 (a competitive CaMKII inhibitor) (Figure 5). Although our immunofluorescence results for CaMKII and NR2B (Supplementary Figure 4) indicated that most cells exhibited neuronal morphology, it remains unclear whether expression of p-NR2B and p-CaMKIIα in the LHb is neuron-specific.

Activation of the NR2B subunit induces a postsynaptic increase in Ca^2+^ influx, ultimately resulting in a sustained excitatory postsynaptic potential, which may in turn lead to LTP via the CaMKII pathway (Noh and Ismail, 2020; Uniyal et al., 2022a). This process is among the main neuronal mechanisms involved in chronic pain. In our study, injection of NMDA into the LHb increased the expression of p-NR2B and p-CaMKII in naive mice, while injection of MK801 into the LHb down-regulated the expression of p-CaMKII in pT-ION mice (Supplementary Figure 3). These results demonstrate that neurons of the LHb may undergo plasticity changes and central sensitization after pT-ION.

Previous studies have indicated that anxiety is regulated by intervening activity in the bilateral LHb (Chan et al., 2017; Purvis et al., 2018; Li et al., 2019). Our previous study demonstrated that inhibiting the excitability of bilateral LHb glutaminergic (CaMKII^+^) neurons significantly attenuates pT-ION-induced anxiety-like behaviors (Cui et al., 2020b). Our results are consistent with those of previous studies showing that bilateral activation of LHb glutaminergic neurons induces anxiety-like behaviors similar to those induced by pT-ION. A prior rat study reported that expression of c-Fos, a marker of neuronal activation, is obviously elevated in the LHb during pain stimulation (Lehner et al., 2004). Other studies have indicated that injection of morphine into the LHb activates μ opioid receptors, while injection of lidocaine inhibits neuronal excitability, which can significantly relieve TN in rats (Khalilzadeh and Saiah, 2017). In this study, bilateral activation of LHb glutaminergic neurons via an HM3Dq-CNO intervention also induced orofacial allodynia similar to that induced by pT-ION. The activation of CaMKII^+^ neurons in the bilateral LHb may induce pain and anxiety via LHb regulation of the 5-HT, dopaminergic (Li et al., 2017; Metzger et al., 2021), and overall reward systems (Wang et al., 2017) given its complex network of nerve projections (Hetu et al., 2016; Browne et al., 2018). Although adult male C57BL/6 mice are frequently used in the study of TN and related emotions (Trevisan et al., 2016; Chen et al., 2021) and we also provided preliminary evidence demonstrating that the LHb plays an important role in the regulation of TN and related anxiety-like behaviors using these mice (Cui et al., 2020b), whether our findings differs according to species and sex requires further validation. This is an important topic that we are currently working on.

## Conclusion

The current results demonstrate that ION injury induces chronic orofacial pain and anxiety-like behaviors in mice. This process may be associated with the activation of NMDA/CaMKII signaling and the hyperexcitation of CaMKII^+^ neurons in the LHb. Bilateral injection of NMDAR and CaMKII inhibitors into the LHb markedly attenuated TN and related anxiety-like behaviors in model mice. Importantly, the orofacial pain and anxiety-like behavior induced by pT-ION could be mimicked by injecting an NMDAR agonist and HM3Dq-CNO into the LHb. Therefore, selective inhibition of LHb function by targeting NMDA/CaMKII signaling may represent a therapeutic approach to pain and related experiences of anxiety in patients with TN.

## Data availability statement

The original contributions presented in this study are included in the article/Supplementary material, further inquiries can be directed to the corresponding author/s.

## Ethics statement

The animal study was reviewed and approved by the Experimental Animal Management Committee of the Affiliated Hospital of Shandong University of Traditional Chinese Medicine.

## Author contributions

W-QC, X-QX, and Z-FZ: study design and planning. Z-FZ, H-YW, Y-YS, LL, XC, and JY: study conduct. W-QC and X-QX: technical guidance. Z-FZ, H-YW, Y-YS, and LL: data analysis. Z-FZ, H-YW, and Y-YS: writing manuscript. All authors reviewed the manuscript and approved the submitted version.
