# Supplementary material for: N-Methyl D-aspartate receptor subtype 2B/Ca2+/calmodulin-dependent protein kinase II signaling in the lateral habenula regulates orofacial allodynia and anxiety-like behaviors in a mouse model of trigeminal neuralgia
Source: Front Cell Neurosci. 2022 Sep 14;16:981190. doi: 10.3389/fncel.2022.981190 (PMC9521491; doi:10.3389/fncel.2022.981190)
Supplement: Supplementary file 1 [file Presentation_1.pdf]

Supplementary Information for

N-Methyl D-aspartate receptor subtype 2B/Ca2+/calmodulin-dependent protein kinase II signaling in the lateral habenula regulates orofacial allodynia and anxiety-like behaviors in a mouse model of trigeminal neuralgia

Methods

Rotarod test

The rotarod test was performed to determine whether injection of MK801 into the LHb influenced locomotor function. Under controlled experimental conditions, the mice were placed on the rotarod (ENV-575M, Med Associates, USA), which was slowly accelerated from a speed of 4 rpm to 24 rpm over 180 s. Rotarod CUB software was used to record the time required for the mouse to fall from the rotarod within this period. The test was repeated three times for each mouse, with a 120-s break between trials. The mean value was used for further analyses. On the day before the evaluation, the mouse was adaptively trained three times with a 30-min break between trials.

Immunofluorescence

According to the method of main text, The brain samples were managed and sectioned into 30 μm-thick slices using a freezing microtome (Leica 2000, Germany). After washing with PBST (PBS solution with 0.3% Triton X-100) three times and then blocked with 5% donkey serum for 2.0 hours at room temperature. Then the slices were incubated with primary antibodies: NR2B (1:1000 dilution, ab28373, Abcam, UK) and CaMKII (1:1000 dilution, 50049, Cell signaling technology, USA) overnight at 4°C . The sections were then washed with PBST (PBS solution with 0.3% Triton X-100) three times and incubated with Alexa 594-conjugated secondary antibody (1:1,000 dilution; Invitrogen, Carlsbad, CA) for 1.5 hour at room temperature. The IF images were captured using a multiphoton laser dot scanning confocal microscope system (FV1000; Olympus, Japan).

Figure legends

Figure S1. The time course of changes in NR2B, p-NR2B, CaMKII, and p-CaMKII

expression after pT-ION modeling. WB revealed no significant differences in the expression of NR2B or CaMKII at day 7, 14, or 21 after pT-ION modeling (NR2B: one-way ANOVA and Dunnett's test, F = 0.1446, $p = 0.9312$, Figure S1A and B; CaMKII: one-way ANOVA and Dunnett's test, F = 0.8306, $p = 0.5023$, Figure S1A and C), when compared with levels of expression in the naive group. Significant increases in p-NR2B and p-CaMKII expression were observed at day 14 and day 21 after pT-ION, but not at day 7 (p-NR2B: one-way ANOVA and Dunnett's test, F = 24.88, $p < 0.0001$, Figure S1C and D; p-CaMKII: one-way ANOVA and Dunnett's test, F = 8.987, $p = 0.0021$, Figure S1C and E). N = 4 mice per group, $^{*}p < 0.05$, $^{**}p < 0.01$, $^{***}p < 0.001$ vs. the Naive group.

Figure S2. Orofacial pain threshold and motor ability after MK801 injection in naive mice. Injection of the NMDA antagonist MK801 (2 μg, 150 nL, S2876, Selleck, USA) into the LHb did not affect the orofacial mechanical pain threshold (von Frey Test-V2: one-way ANOVA and Dunnett's test, F = 0.0395, $p = 0.9613$, Figure S2A; von Frey Test-V3: one-way ANOVA and Dunnett's test, F = 0.1382, $p = 0.8717$, Figure S2B), cold pain threshold (one-way ANOVA and Dunnett's test, F = 0.0397, $p = 0.9611$, Figure S2C), or motor ability (one-way ANOVA and Dunnett's test, F = 0.0519, $p = 0.9495$, Figure S2D) in naive mice. N = 8 mice per group.

Figure S3. Expression of p-CaMKII at 4 hours after MK801 injection. WB revealed that p-CaMKII expression was significantly decreased in mice injected with MK801 when compared with that in the pT-ION and DMSO groups (one-way ANOVA and Tukey's test, F = 20.17, $p = 0.0005$, Figure S3A and C), whereas the levels of CaMKII had no significant changes in the MK801 injection group (one-way ANOVA and Tukey's test, F = 0.955, $p = 0.4206$, Figure S3A and B). N = 4 per group, $^{**}p < 0.01$, $^{***}p < 0.001$.

Figure S4. Immunofluorescence staining for CaMKII and NR2B in the LHb. Double immunostaining of NR2B (red) and DAPI (blue) (A). Double immunostaining of CaMKII (red)

and DAPI (blue) (B). Scale bar: 100 μm.
