# Supplementary figures and images for: N-Methyl D-aspartate receptor subtype 2B/Ca2+/calmodulin-dependent protein kinase II signaling in the lateral habenula regulates orofacial allodynia and anxiety-like behaviors in a mouse model of trigeminal neuralgia
Source: Front Cell Neurosci. 2022 Sep 14;16:981190. doi: 10.3389/fncel.2022.981190 (PMC9521491; doi:10.3389/fncel.2022.981190)

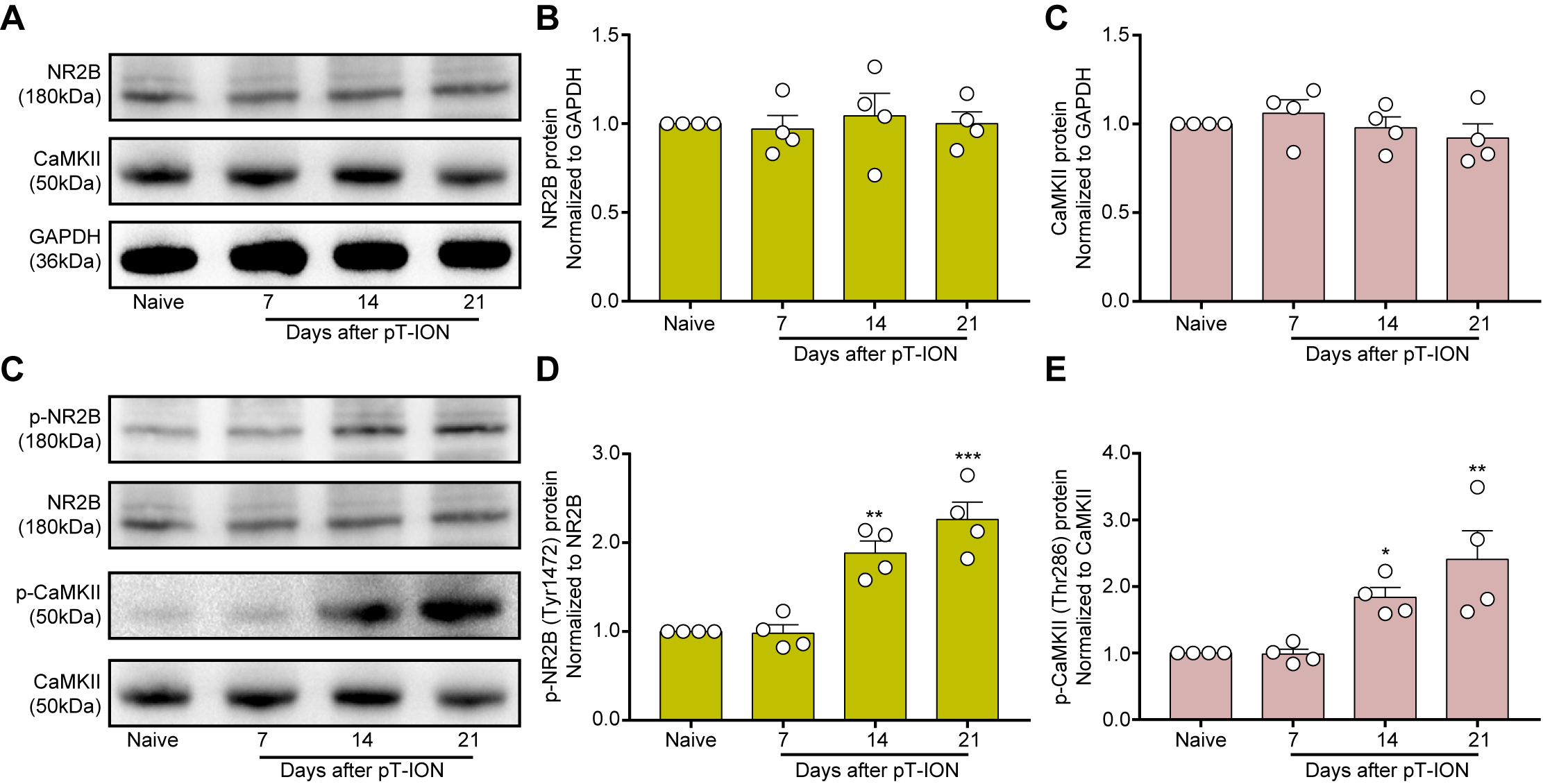

Supplement: Supplementary file 2 [file Image_1.TIF]

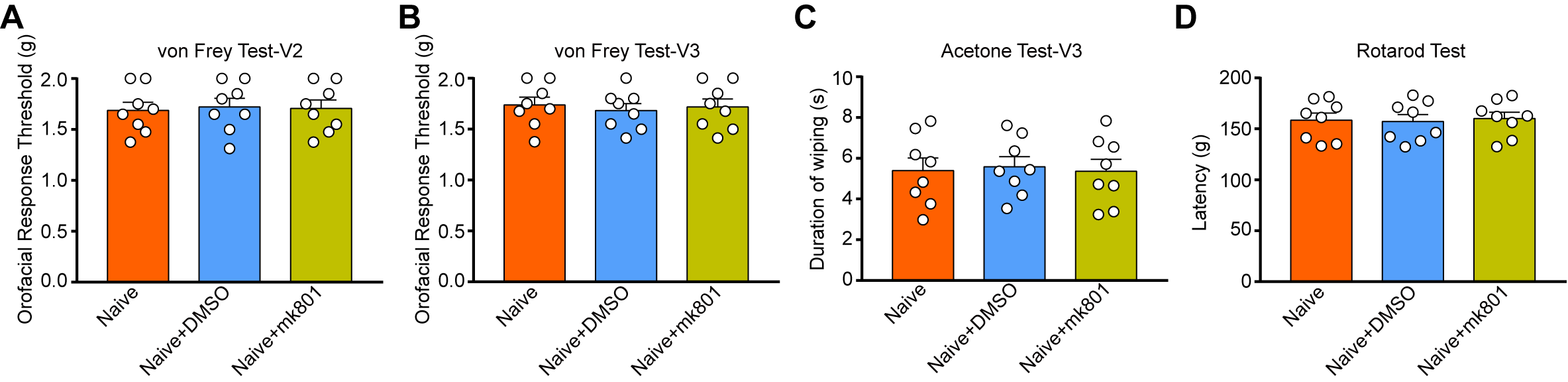

Supplement: Supplementary file 3 [file Image_2.TIF]

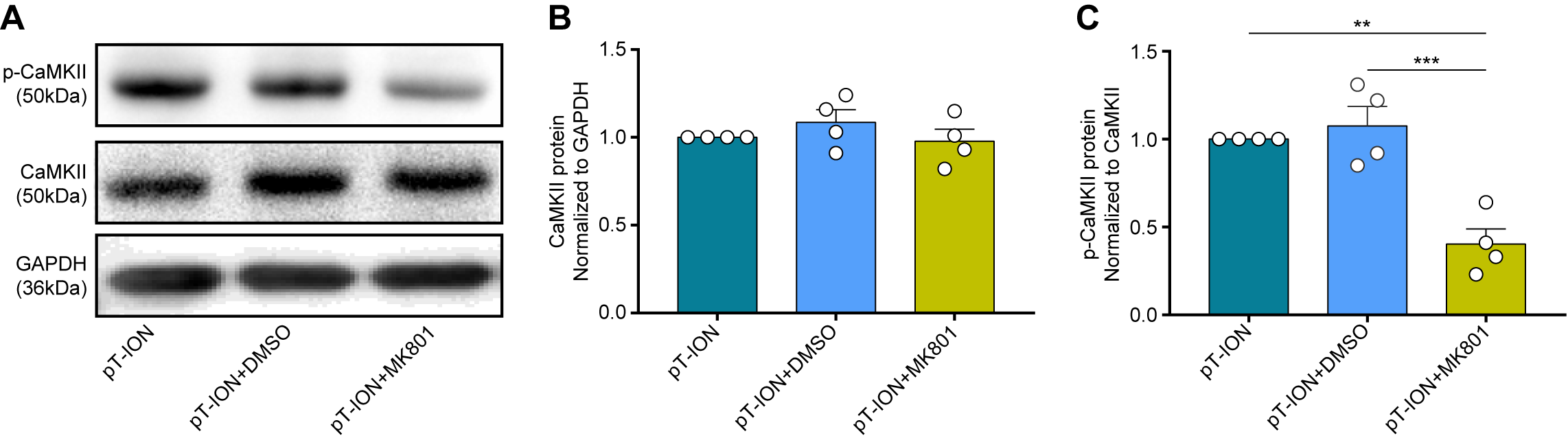

Supplement: Supplementary file 4 [file Image_3.TIF]

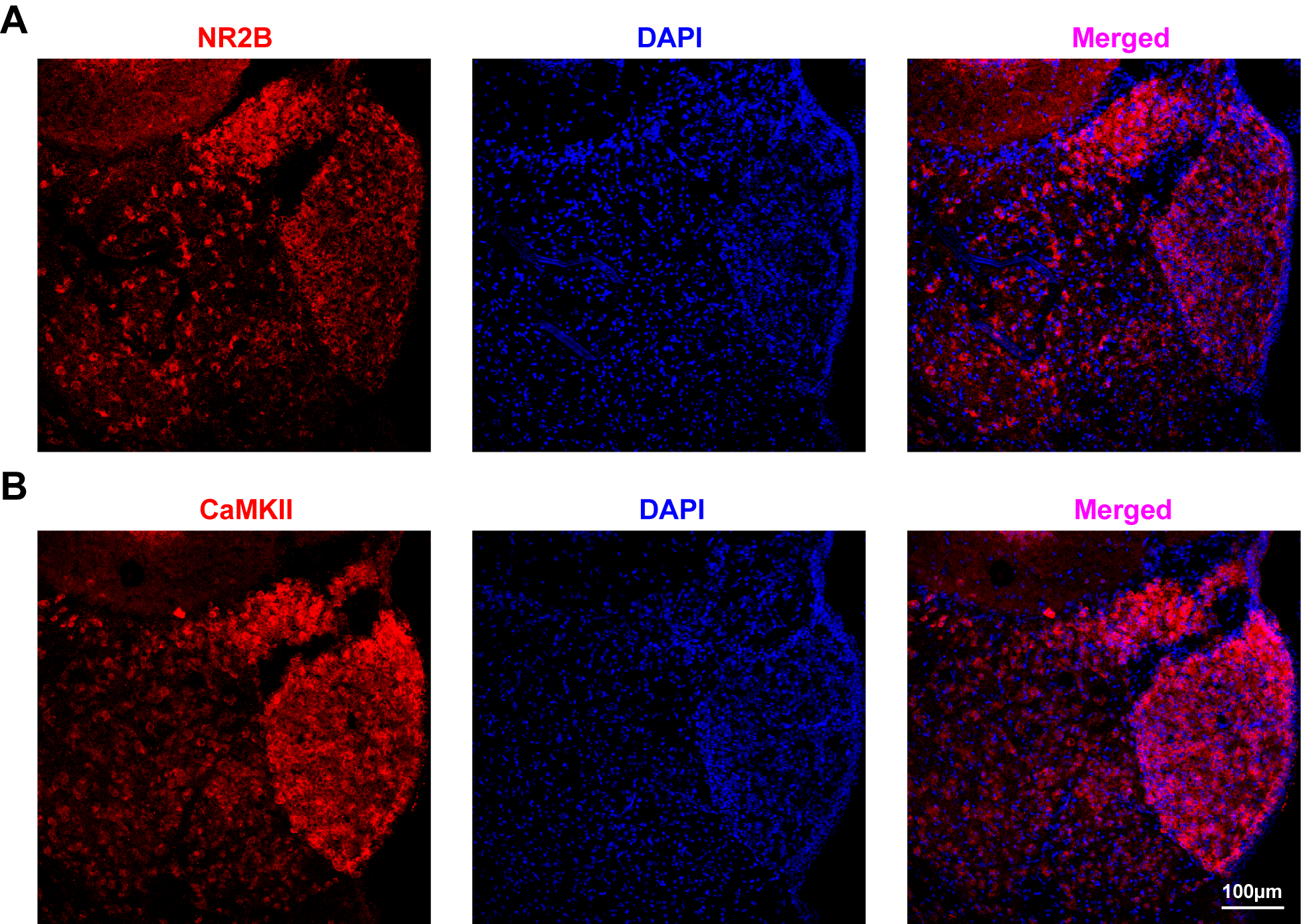

Supplement: Supplementary file 5 [file Image_4.TIF]
